# Meeting Report: Fungal ITS Workshop (October 2012)

**DOI:** 10.4056/sigs.3737409

**Published:** 2013-04-15

**Authors:** Scott T. Bates, Steven Ahrendt, Holly M. Bik, Thomas D. Bruns, J. Gregory Caporaso, James Cole, Michael Dwan, Noah Fierer, Dai Gu, Shawn Houston, Rob Knight, Jon Leff, Christopher Lewis, Juan P. Maestre, Daniel McDonald, R. Henrik Nilsson, Andrea Porras-Alfaro, Vincent Robert, Conrad Schoch, James Scott, D. Lee Taylor, Laura Wegener Parfrey, Jason E. Stajich

**Affiliations:** 1 Cooperative Institute for Research in Environmental Sciences, University of Colorado, Boulder, Colorado, USA; 2 Department of Plant Pathology & Microbiology, University of California Riverside, Riverside, California, USA; 3 UC Davis Genome Center, University of California Davis, Davis, California, USA; 4 Department of Plant and Microbial Biology, University of California Berkeley, Berkeley, California, USA; 5 Department of Computer Science, Northern Arizona University, Flagstaff, Arizona, USA and Argonne National Labs, Argonne, Illinois, USA; 6 Department of Microbiological and Molecular Genetetics, Michigan State University, East Lansing, Michigan, USA; 7 Center for Microbiomics and Human Health, TGen North, Flagstaff, Arizona, USA; 8 Minnesota Supercomputing Institute, University of Minnesota, Minneapolis, Minnesota, USA; 9 Department of Chemistry and Biochemistry, University of Colorado, Boulder, Colorado, USA; 10 Howard Hughes Medical Institute, Boulder, Colorado, USA; 11 Eastern Cereals and Oilseed Research Centre, Agriculture and Agri-food Canada, Ottawa, Ontario, Canada; 12 Department of Civil, Architecture and Environmental Engineering, University of Texas at Austin, Austin, Texas, USA; 13 Department of Chemistry and Biochemistry, and Biofrontiers Institute, University of Colorado, Boulder, Colorado, USA; 14 Department of Biological and Environmental Sciences, University of Gothenburg, Göteborg, Sweden; 15 Department of Biological Sciences, Western Illinois University, Macomb, Illinois, USA; 16 Centraalbureau voor Schimmelcultures- Royal Netherlands Academy of Arts and Sciences, Utrecht, The Netherlands; 17 National Center for Biotechnology Information, National Library of Medicine, National Institutes of Health, Bethesda, Maryland, USA; 18 Division of Occupational & Environmental Health, Dalla Lana School of Public Health, University of Toronto, Toronto, Ontario, Canada; 19 Institute of Arctic Biology, University of Alaska, Fairbanks, Alaska, USA

## Abstract

This report summarizes a meeting held in Boulder, CO USA (19–20 October 2012) on fungal community analyses using ultra-high-throughput sequencing of the internal transcribed spacer (ITS) region of the nuclear ribosomal RNA (rRNA) genes. The meeting was organized as a two-day workshop, with the primary goal of supporting collaboration among researchers for improving fungal ITS sequence resources and developing recommendations for standard ITS primers for the research community.

## ITS sequence database for fungal community analyses

Sequencing-based techniques have allowed characterization of microbial communities from environmental samples without relying on cultivation. Because of improvements in these techniques and of methods required to analyze the resulting datasets, microbial ecology has been advancing rapidly: microbial diversity can be surveyed to an extent previously unimaginable. The internal transcribed spacer (ITS) and other ribosomal RNA gene sequence regions (“rRNA” in the rest of this document) have been used successfully to profile fungal communities using Sanger [1] and 454 [2] sequencing. The arrival of ultra-high-throughput sequencing platforms [3] promise to offer new insights into the diversity and ecology of fungal communities, yet few studies of fungal communities have employed this technology successfully. Progress has been hampered, in part, by the lack of a high-quality reference database of fungal sequences for the ITS region of the rRNA operon [4], which is now the most widely sequenced DNA region in fungi [5] and is the marker of choice for molecular identification of most fungal taxa [6].

For *Bacteria* and *Archaea*, where the ribosomal small subunit (SSU/16S) gene is the primary marker in environmental sequencing, efforts have been made to improve the quality of the public reference sequence datasets, including GreenGenes [7] and RDP [8]. The same is true for more general SSU/large subunit (LSU) rRNA gene sequence databases, such as SILVA, which includes all three domains of life [9]. For fungi, a “hand-curated” LSU sequence reference set is currently available, and work is underway to apply similar methods to improve the ITS database [10]. Because sequencing technologies continue to improve, the number of ITS sequences in primary sequence repositories such as INSDC will steadily increase, and quality control via hand-curation for specialized, publically available rRNA gene sequence databases will not be sustainable.

Primary sequence repositories are already experiencing explosive growth in the number of unidentified environmental fungal ITS sequences [11], yet these sequences will be of limited use in improving our overall understanding of fungal diversity unless they are properly identified and can be placed within a phylogenetic context. When sufficiently closely related sequences exist, environmental sequences can be placed within a phylogenetic context today simply by aligning with related sequences and constructing trees. However, because ITS evolves rapidly, constructing phylogenies that span large taxonomic ranges remains extremely challenging. An even more important problem is that of misidentified sequences (environmental sequences included) currently in public databases. These can lead to erroneous placement of unknowns, even if tree-based approaches are used. Therefore, identifying these errors and re-annotating in an automated fashion is a critical challenge, especially for extremely large datasets where manual phylogenetic analysis is not feasible due to the presence of millions of sequence reads that correspond to “unknown” operational taxonomic units (OTUs). Consequently, clean reference databases as well as automated phylogenetic assignment and analysis methods are critical needs.

## Purposes of the Meeting

**This meeting was organized in order to:**
- Facilitate communication, potential data exchange, and collaboration with the aim of improving fungal ITS sequence resources for the research community.
- Identify suitable ITS primers for fungal community analyses using ultra-high-throughput sequencing.
- Develop strategies for automated (and manual) database curation as well as the naming of environmental sequences and OTUs at various levels of resolution.
- Establish a sustainable plan for reference database development and maintenance.

## Participants

The meeting participants included researchers representing publicly available databases that contain microbial sequence data (e.g., GenBank, GreenGenes, RDP, SILVA) or fungi-specific resources (e.g., MycoBank, UNITE), as well as researchers currently using ultra-high-throughput sequencing to examine fungal communities or those involved in developing software, such as QIIME [12] and PhyloSift [13], to facilitate such studies.

## Activities

The meeting was conducted as a two-day workshop. The first day was devoted primarily to brief presentations by participants outlining their involvement in curating public sequence databases, developing high-throughput sequencing pipelines, or using ultra-high-throughput sequencing to examine fungal diversity in environmental samples (e.g., air or soil). The presentations are available online [14]. The second day focused on discussions related to the assembly of a high-quality reference database of fungal ITS sequences, selecting ITS primers suitable for ultra-high-throughput sequencing, as well as methods to link ITS sequences to the fungal phylogeny for automated curation, quality control, and phylogeny-based community analysis methods.

## Conclusions / Outcomes

Ultra-high-throughput sequence processing/analytical pipelines, such as those implemented in QIIME, rely on de-replication of large sequence datasets through clustering for the creation of reference sequence sets that can be used to assist in the recognition of OTUs from environmental samples. The meeting participants largely supported the use of the UNITE database [15,16] as a focal point for the development of high quality fungal ITS reference sequence sets. UNITE currently has implemented several desirable features for this task, including:

- A comprehensive set of approximately 300,000 fungal ITS sequences extracted from public databases.
- An annotation management system (*PlutoF*) that allows qualified third-party users to add pertinent metadata (e.g., on ecology or geography), improve the taxonomic resolution, tag problematic entries, or correct misidentifications for sequences in the UNITE database.
- *Global Key Annotations* that permit visualization of sequences clustered at a range of similarity levels (99–97%), with the sequences representing each OTU in the cluster depicted in an alignment.
- Cluster centroids selected with a preference toward using sequences that are reliable (e.g., generated from trustworthy sources such as the *Assembling the Fungal Tree of Life* project or hand-picked by taxonomic experts), taxonomically informative (e.g., identified to the species-level), or that have particular relevance for taxonomy/nomenclature (e.g., sequences from type specimens).
- Improvements on the horizon (slated for early 2013) included labeling sequences representing cluster centroids that will allow these unique sequences to be tracked through time as clusters change, as well as options for downloading cluster centroid sequence sets for different sequence similarity levels.

With the availability of these cluster centroids, reference sequence sets for ultra-high-throughput pipelines can be directly generated from the UNITE database in a rapid manner. The meeting allowed for coordination that resulted in the creation of an alpha version of the UNITE reference set to facilitate OTU picking and taxonomic assignment for fungal ITS sequence reads generated in high-/ultra-high-throughput sequencing runs. This reference set is now publically available on the QIIME website [12,17].

UNITE currently provides taxonomic strings based on classification schema culled from fungal taxonomic resources such as Index Fungorum [18] and MycoBank [19], which are comprehensive databases for fungal names that offer expertise and resources for improving the quality and availability of fungal taxonomic information. Journals publishing novel fungal taxa now typically require authors to register new names in MycoBank, which in turn is encouraging submission of informative DNA sequences, such as the ITS region, associated with new taxa to the public databases. In addition to acting as sequence vouchers for type material, these data also have the potential to inform molecular studies examining environmental samples.

Synergistic collaboration and the flow of information between, and within, online taxonomic resources and the public sequence databases (that have expressed interest in using a global standardized taxonomy) were seen by meeting participants as being highly desirable. The integration of fungal taxonomy and phylogeny was deemed another important consideration. The *Assembling the Fungal Tree of Life* (AFToL) project made considerable progress toward refining our understanding of the fungal phylogeny, which informed taxonomy for the kingdom [20]. Sequences generated under AFToL represent reliable data that are desirable for cluster centroids in the fungal reference sets. New projects, such as the *Open Tree of Life* [21], hold potential as phylogeny-based taxonomic resources for reference databases, while others, such as *Fungal Barcoding* [22], promise to produce additional high-quality sequences across a wide range of fungal groups for improving the reference datasets.

Although the ITS marker allows fungal sequences to be resolved at the genus- or species-level, aligning ITS sequences across a wide taxonomic range is essentially unworkable. Thus, meeting participants also discussed strategies for anchoring fungal ITS sequences to the broader phylogeny (e.g., one constrained to the AFToL backbone) using SSU or LSU sequences having contiguous ITS reads. Given that for fungi SSU (18S) is typically uninformative below the order- or family-level, LSU (26/28S) was seen as an appropriate marker for this task. The large subunit rRNA gene was considered desirable, as it has been extensively used in fungal phylogenetic studies, it allows for accurate placement in the phylogeny both at higher and lower taxonomic ranks (e.g., phylum/class and family/genus, respectively), and many reliable AFToL sequences (spanning both LSU and the ITS region) are currently available for this purpose [10,23]. Other reliable sequence sources for full-length LSU/ITS reads were identified from complete genomes, such as fungal genomes project [24]. With the anchor tree established, fungal ITS reads produced by ultra-high-throughput sequencing can potentially be placed within a phylogenetic context for taxonomic validation, improving the taxonomy associated with unknown environmental sequences, identifying and naming novel environmental groups, as well as for other automated curation tasks. Linking ITS reads to the fungal phylogeny will also allow for phylogenetic metrics of community distances (e.g., UniFrac) [25] to be used in beta-diversity analyses.

Two talks on the first day of the workshop presented preliminary data from ultra-high-throughput sequence surveys of soil fungi targeting the ITS1 region using the primer pairs ITS1-F/ITS2 [see 26]. Due to spliceosomal inserts known to exist toward the 3′ end of SSU rRNA gene that could interfere with priming sites and cause biases against groups of fungi (e.g., *Helotiales*) where such inserts exits, participants expressed a preference for using primers for the ITS2 region, for which such spliceosomal inserts are not known. Additional reasons for adopting the use of the ITS2 region marker include its close proximity to LSU (e.g., for anchoring to the phylogeny, see above), less variation in read length compared to ITS1, and the availability of data on ITS2 secondary structure [27,28] that can inform sequence alignments. With continually improving read lengths of ultra-high-throughput sequencing platforms, full length ITS/LSU reads may be possible in the near future. Primers targeting the ITS2 region have been identified, and are currently being tested for use in ultra-high-throughput sequencing. Recommendations for fungal ITS2 as well as current versions of the UNITE centroid reference sequence sets will be available on the QIIME website [12] in the coming year.

## References

[1] O’Brien HE Parrent JL Jackson JA Moncalvo JM Vilgalys R Fungal community analysis by large-scale sequencing of environmental samples. Appl Environ Microbiol 2005; 71:5544-5550 10.1128/AEM.71.9.5544-5550.2005 16151147 PMC1214672

[2] Jumpponen A Jones KL Massively parallel 454 sequencing indicates hyperdiverse fungal communities in temperate *Quercus macrocarpa* phyllosphere. New Phytol 2009; 184:438-448 10.1111/j.1469-8137.2009.02990.x 19674337

[3] Caporaso JG Lauber CL Walters WA Berg-Lyons D Huntley J Fierer N Betley J Fraser L Bauer M Gormley N Ultra-high-throughput microbial community analysis on the Illumina HiSeq and MiSeq platforms. ISME J 2012; 6:1621-1624 10.1038/ismej.2012.8 22402401 PMC3400413

[4] Nilsson RH Tedersoo L Abarenkov K Ryberg M Kristiansson E Hartmann M Schoch CL Nylander JAA Bergsten J Porter TM Five simple guidelines for establishing basic authenticity and reliability of newly generated fungal ITS sequences. MycoKeys 2012; 4:37-63 10.3897/mycokeys.4.3606

[5] Peay KG Kennedy PK Bruns TD Fungal community ecology: a hybrid beast with a molecular master. Bioscience 2008; 58:799-810 10.1641/B580907

[6] Schoch CL Seifert KA Huhndorf S Robert V Spouge JL Levesque CA Chen W Nuclear ribosomal internal transcribed spacer (ITS) region as a universal DNA barcode marker for fungi. Proc Natl Acad Sci USA 2012; 109:6241-6246 10.1073/pnas.1117018109 22454494 PMC3341068

[7] McDonald D Price MN Goodrich J Nawrocki EP DeSantis TZ Probst A Andersen GL Knight R Hugenholtz P An improved Greengenes taxonomy with explicit ranks for ecological and evolutionary analyses of bacteria and archaea. ISME J 2012; 6:610-618 10.1038/ismej.2011.139 22134646 PMC3280142

[8] Cole JR Wang Q Cardenas E Fish J Chai B Farris RJ Kulam-Syed-Mohideen AS McGarrell DM Marsh T Garrity GM Tiedje JM The Ribosomal Database Project: improved alignments and new tools for rRNA analysis. Nucleic Acids Res 2009; 37:D141-D145 10.1093/nar/gkn879 19004872 PMC2686447

[9] Pruesse E Quast C Knittel K Fuchs BM Ludwig W Peplies J Glöckner FO SILVA: a comprehensive online resource for quality checked and aligned ribosomal RNA sequence data compatible with ARB. Nucleic Acids Res 2007; 35:7188-7196 10.1093/nar/gkm864 17947321 PMC2175337

[10] Liu KL Kuske CR Porras-Alfaro A Eichorst S Xie G Accurate, rapid taxonomic classification of fungal large subunit rRNA genes. Appl Environ Microbiol 2012; 78:1523-1533 10.1128/AEM.06826-11 22194300 PMC3294464

[11] Hibbett DS Ohman A Glotzer D Nuhn M Kirk P Nilsson RH Progress in molecular and morphological taxon discovery in Fungi and options for formal classification of environmental sequences. Fungal Biol Rev 2011; 25:38-47 10.1016/j.fbr.2011.01.001

[12] QIIME http://qiime.org

[13] PhyloSift http://phylosift.wordpress.com

[14] http://fungalgenomes.org/public/Fungi_ITS/Oct_2012_Boulder.

[15] Abarenkov K Nilsson RH Larsson KH Alexander IJ Eberhardt U Erland S Høiland K Kjøller R Larsson E Pennanen T The UNITE database for molecular identification of fungi: recent updates and future perspectives. New Phytol 2010; 186:281-285 10.1111/j.1469-8137.2009.03160.x 20409185

[16] UNITE database. http://unite.ut.ee

[17] http://qiime.org/home_static/dataFiles.html.

[18] Index Fungorum http://www.indexfungorum.org

[19] MycoBank http://www.mycobank.org

[20] Hibbett DS Binder M Bischoff JF Blackwell M Cannon PF Eriksson OE Huhndorf S James T Kirk PM Lücking R A higher-level phylogenetic classification of the Fungi. Mycol Res 2007; 111:509-547 10.1016/j.mycres.2007.03.004 17572334

[21] *Open Tree of Life* http://opentreeoflife.org

[22] *Fungal Barcoding* http://www.fungalbarcoding.org

[23] Bruns TD Vilgalys R Barnes SM Gonzalez D Hibbett DS Lane DJ Simon L Stickel S Szaro TM Weisburg WG Sogin ML Evolutionary relationships within the fungi: analyses of nuclear small subunit rRNA sequences. Mol Phylogenet Evol 1992; 1:231-241 10.1016/1055-7903(92)90020-H 1342940

[24] 1000 Fungal Genoms Project. http://1000.fungalgenomes.org

[25] Lozupone C Hamady M Knight R UniFrac: An Online Tool for Comparing Microbial Community Diversity in a Phylogenetic Context. BMC Bioinformatics 2006; 7:371 10.1186/1471-2105-7-371 16893466 PMC1564154

[26] Bellemain E Carlsen T Brochmann C Coissac E Taberlet P Kauserud H ITS as an environmental DNA barcode for fungi: an *in silico* approach reveals potential PCR biases. BMC Microbiol 2010; 10:189 10.1186/1471-2180-10-189 20618939 PMC2909996

[27] Koetschan C Förster F Keller A Schleicher T Ruderisch B Schwarz R Müller T Wolf M Schultz J The ITS2 Database III: sequences and structures for phylogeny. Nucleic Acids Res 2010; 38:D275-D279 10.1093/nar/gkp966 19920122 PMC2808966

[28] The ITS2 Database III http://its2.bioapps.biozentrum.uni-wuerzburg.de

